# Visual field bias in hearing and deaf adults during judgments of facial expression and identity

**DOI:** 10.3389/fpsyg.2013.00319

**Published:** 2013-06-06

**Authors:** Susan M. Letourneau, Teresa V. Mitchell

**Affiliations:** ^1^Department of Psychology, Brandeis UniversityWaltham, MA, USA; ^2^Consortium for Research and Evaluation of Advanced Technologies in Education, Department of Educational Communication and Technology, New York UniversityNew York, NY, USA; ^3^Department of Educational Psychology, The Graduate Center, City University of New YorkNew York, NY, USA; ^4^Department of Psychiatry, Eunice Kennedy Shriver Center, University of Massachusetts Medical SchoolWaltham, MA, USA

**Keywords:** deafness, face perception, visual field bias, laterality, sign language, emotional expression

## Abstract

The dominance of the right hemisphere during face perception is associated with more accurate judgments of faces presented in the left rather than the right visual field (RVF). Previous research suggests that the left visual field (LVF) bias typically observed during face perception tasks is reduced in deaf adults who use sign language, for whom facial expressions convey important linguistic information. The current study examined whether visual field biases were altered in deaf adults whenever they viewed expressive faces, or only when attention was explicitly directed to expression. Twelve hearing adults and 12 deaf signers were trained to recognize a set of novel faces posing various emotional expressions. They then judged the familiarity or emotion of faces presented in the left or RVF, or both visual fields simultaneously. The same familiar and unfamiliar faces posing neutral and happy expressions were presented in the two tasks. Both groups were most accurate when faces were presented in both visual fields. Across tasks, the hearing group demonstrated a bias toward the LVF. In contrast, the deaf group showed a bias toward the LVF during identity judgments that shifted marginally toward the RVF during emotion judgments. Two secondary conditions tested whether these effects generalized to angry faces and famous faces and similar effects were observed. These results suggest that attention to facial expression, not merely the presence of emotional expression, reduces a typical LVF bias for face processing in deaf signers.

## Introduction

Multiple neural pathways process the information contained in faces. Cortical regions involved in face perception include the fusiform gyrus (FG), which processes invariant aspects of faces, such as identity and gender, and the superior temporal sulcus (STS), which processes changeable aspects of faces, such as expression and directional gaze (Haxby et al., 2002). Activation of these cortical areas is typically right lateralized in response to face stimuli, with tendencies toward larger and faster responses in the right hemisphere than the left (Bentin et al., 1996; Rossion et al., 2000). Right lateralized activity in response to faces is also associated with a processing advantage for face information in the left visual field (LVF) as opposed to the right visual field (RVF). This bias results in relatively higher accuracies in face perception tasks for stimuli presented in the LVF than the RVF (Yovel et al., 2008). Hemispheric and visual field laterality is therefore considered a feature of normal face perception in typically developing adults.

The establishment of this neural network develops well into adolescence, becoming gradually more focal and lateralized with age (Itier and Taylor, 2004; Taylor et al., 2004; Passarotti et al., 2007). The protracted development of neural mechanisms of face perception raises the possibility that atypical visual experience with faces early in life might alter the development of the face perception system. This hypothesis has been applied to studies of face perception in autistic individuals, who demonstrate atypical gaze patterns when viewing faces (Pelphrey et al., 2002; Jones et al., 2008; Rutherford and Towns, 2008), impaired holistic processing of faces (Teunisse and De Gelder, 2003), and reduced activation of cortical regions involved in face perception (Schultz et al., 2000), although this hypoactivation may be driven by atypical scanning patterns on faces (Hadjikhani et al., 2004; Dalton et al., 2005).

Subtle changes in the development of face perception are observed in other atypical populations that do not demonstrate the social dysfunctions observed in autism. In particular, profound deafness and experience with sign language are not associated with face perception dysfunctions—in fact, deaf signers have been shown to perform more accurately than hearing adults on the Benton test of face recognition (McCullough and Emmorey, 1997). Nevertheless, deafness and sign language use place unique functional pressures on face perception (Mitchell and Maslin, 2007). For deaf users of American Sign Language (ASL), facial expressions convey not only emotion information, but also a variety of linguistic information that would otherwise be conveyed by one's pitch or tone of voice in a spoken language (see Baker-Shenk, 1985; Corina et al., 1999 for reviews). Accurate comprehension of ASL requires the perception of rapid changes in facial expression, which can serve both grammatical functions [e.g., indicating a question, or the boundaries of a relative clause; (Baker-Shenk, 1985; Corina et al., 1999)] and semantic functions [e.g., as adverbs, indicating manner of action; (Anderson and Reilly, 1998)].

For deaf signers, the integration of face and language processing may influence the hemispheric and visual field biases observed during face perception tasks. Previous studies have revealed that the LVF bias typically observed during face perception was eliminated or reversed in deaf signers, specifically during the perception of linguistic facial expressions (Corina, 1989; Corina et al., 1999). Other studies have found reductions in RVF biases observed during emotional face perception (Vargha-Khadem, 1983; Szelag and Wasilewski, 1992; Szelag et al., 1992), although these effects were sometimes dependent on task order or instructions (Corina, 1989). Specifically, Corina (1989) found that LVF biases were reduced when subjects completed a linguistic task prior to an emotion task, suggesting a priming effect following exposure to linguistic stimuli. The author concluded that for deaf signers, the visual field biases observed during face perception tasks may depend on the specific type of information being gathered from the face. This finding suggests that visual field biases in deaf signers may be dynamic, meaning that they may vary based on the type of face stimuli presented and/or the demands of the task.

Recent neuroimaging studies of deaf signers have reported reductions in right hemisphere biases in response to expressive faces, although these effects varied depending on whether the faces were shown in isolation or whether they were presented with manual ASL signs, and whether the tasks were linguistic in nature. For example, McCullough et al. (2005) found that hearing non-signers showed bilateral activation in the FG in response to emotional and linguistic facial expressions. The hearing group showed a trend toward right lateralized activation of the STS in response to emotional expressions paired with manual signs, but bilateral activation for other stimulus conditions. For deaf signers, however, FG activation was left lateralized in response to emotional facial expressions. STS activation was bilateral for emotional expressions and for linguistic expressions presented alone, but left lateralized for linguistic expressions that appeared along with a manual ASL verb. These results point to complex interactions between the presence of linguistic information in a face stimulus and the relative recruitment of left vs. right hemisphere structures in deaf signers. In a second study, hearing signers, like deaf signers, demonstrated bilateral activation in the STS in response to emotional expressions paired with an ASL verb (Emmorey and Mccullough, 2009). However, unlike deaf signers, the hearing signers in this study did not show left lateralized STS activity in response to linguistic expressions. These results suggest that both ASL experience and deafness itself contribute to hemisphere biases observed in deaf signers.

In another study (Weisberg et al., 2012), deaf signers judging neutral faces in an identity matching task showed reduced activation in the right FG, but enhanced activation in the right STS, compared to hearing non-signers. Hearing signers in this study showed activation patterns that were “in between” those of hearing non-signers and deaf signers, indicating that a combination of deafness and ASL experience may drive the observed effects. In sum, results of neuroimaging studies of deaf signers suggest that deafness and sign language experience are associated with greater recruitment of the left hemisphere during some face perception tasks, perhaps in part due to the involvement of face perception in ASL communication.

This hypothesis is supported by previous research on other visual processes that are involved in ASL comprehension. For example, the laterality of motion perception is also altered in deaf signers. In motion detection paradigms, hearing adults are more accurate when detecting motion in the left peripheral visual field rather than the right, while both deaf and hearing signers show the opposite laterality (Neville and Lawson, 1987a,b; Bosworth and Dobkins, 1999, 2002b). Studies that measured neural activity using event-related potentials (Neville and Lawson, 1987a,b) and functional magnetic resonance imaging (Bavelier et al., 2001) in response to peripheral moving stimuli have reported larger responses in the right than the left hemisphere for hearing non-signers, but in the left hemisphere for both deaf and hearing signers. Thus, visual processes that are central to ASL perception (such as the perception of motion and expressive faces) may alter visual field asymmetries by increasing the involvement of the left hemisphere.

Although some previous studies have found evidence that the laterality of face perception is altered in deaf signers viewing emotional and linguistic facial expressions, these studies have not made direct comparisons between emotion and identity judgments using comparable stimuli (or other face perception tasks that are not linked to ASL perception). Furthermore, the neuroimaging study conducted by McCullough et al. (2005) used face stimuli that were sometimes accompanied by manual ASL signs, potentially increasing the involvement of the left hemisphere in this task. In a matching task with neutral faces, Weisberg et al. (2012) found no change in left hemisphere activation in deaf signers compared to hearing non-signers, suggesting that attention to expressive aspects of faces may be responsible for eliciting the shifts in visual field bias observed by McCullough et al. (2005).

However, existing evidence cannot determine whether the laterality of face perception in deaf signers varies with stimulus qualities, with task demands, or both. Specifically, are visual field biases in deaf signers similar whenever expressive faces are presented, regardless of whether they are asked to judge these expressions? Alternatively, when the stimuli are constant, do deaf signers show different visual field biases when making different kinds of judgments?

One approach to this research question would be to examine the laterality of face perception when participants judge the identity of expressive faces, when the facial expression is present but not relevant to the task. This approach would allow us to determine whether visual field biases are altered whenever expressive faces are observed, or whether visual field biases vary only with explicit attention to facial expression. Because identity judgments are not tied to ASL perception, the demonstration of shifts in the laterality during identity tasks would provide evidence that fundamental face perception mechanisms, not just those specific to ASL perception, are altered in lifelong signers.

With these issues in mind, the current study required participants to make emotion and identity judgments of an identical set of expressive faces. Participants were first familiarized with a small set of faces, which then served as stimuli for both an emotion task and an identity task. The stimuli in both tasks therefore contained varying identities and expressions, allowing for an examination of the effects of explicit attention to emotional expression on the laterality of face perception in hearing vs. deaf adults. Finally, in order to examine the generalizability of our findings, participants completed an additional emotion condition, in which the same models posed a facial expression different from that posed in the primary emotion condition, and an additional identity condition, in which famous rather than familiar faces were presented.

## Materials and methods

### Subjects

Twelve hearing adults (2 male) aged 21–39 (median age 25), and twelve deaf adults (4 male) aged 23–51 (median age 30) participated in the study. Deaf subjects were severely to profoundly deaf, had been deaf since infancy, and began learning sign language either natively or by the age of 5. Additional information about deaf participants is provided in Table 1. Hearing subjects had no experience with ASL. All subjects self-reported to have no neurological or psychological diagnoses, and had normal or corrected-to-normal vision as assessed by a “Tumbling-E” Snellen-type chart. Subjects provided informed consent under protocols approved by the University of Massachusetts Medical School and Brandeis University IRBs and were paid for their participation. Deaf participants were tested by an experimenter fluent in ASL.

**Table 1:** **Deaf participant demographics**.

**Subject number**	**Deaf family members**	**Cause of deafness**	**Age when diagnosed**	Age of ASL exposure^*^
1	Parents, sister	Genetic	<1 year	Birth
2	Parents, siblings (4th generation deaf family)	Genetic	<1	Birth
3	Parents, siblings (4th generation deaf family)	Genetic	<1	Birth
4	Parents (5th generation deaf family)	Genetic	<1	Birth
5	Older sister	Unknown	2	Birth
6	Hard of hearing sisters	One ear deaf at birth, unknown cause. Mumps at 10 mo. deafened other ear	<1	1
7	None	Kniest dysplasia	<1	1
8	None	Spinal meningitis	1	1
9	None	Unknown	1	1
10	None	Unknown	1.5	Chinese SL: 3, ASL: adult
11	None	Unknown	3	4
12	None	Unknown	1.5	SEE:3, ASL:5

* *Deaf individuals with deaf parents or older siblings were exposed to ASL at home from birth. For deaf individuals without deaf family members, the age at which they began learning ASL is listed. This usually took place at deaf community centers and deaf schools. SEE, Signed Exact English; Chinese SL, Chinese sign language; this participant was fluent in ASL as an adult. All deaf participants used ASL daily as their primary means of communication*.

### Stimuli

Stimuli included sixteen models (eight women and eight men) from the NimStim set (Tottenham et al., 2009) posing various emotional expressions (Neutral, Happy, Angry, Surprised, Disgusted, Sad). Eight of the models (4 women and 4 men) were used in the familiarization training (described below), and were therefore familiar to the subjects before the start of the experimental trials. The remaining eight models were unfamiliar to the subjects.

In addition, images of eight famous celebrities were used for one block of experimental trials. These celebrities included four women (Julia Roberts, Angelina Jolie, Madonna, Reese Witherspoon) and four men (Matt Damon, Tom Cruise, Brad Pitt, George Clooney). The images chosen showed the celebrities directly facing the camera, with closed-mouth happy expressions, and without facial hair or distinguishing accessories (e.g., glasses, jewelry). Images were cropped and edited in Adobe Photoshop to resemble the NimStim images (with the background and clothing concealed).

**Figure 1:**
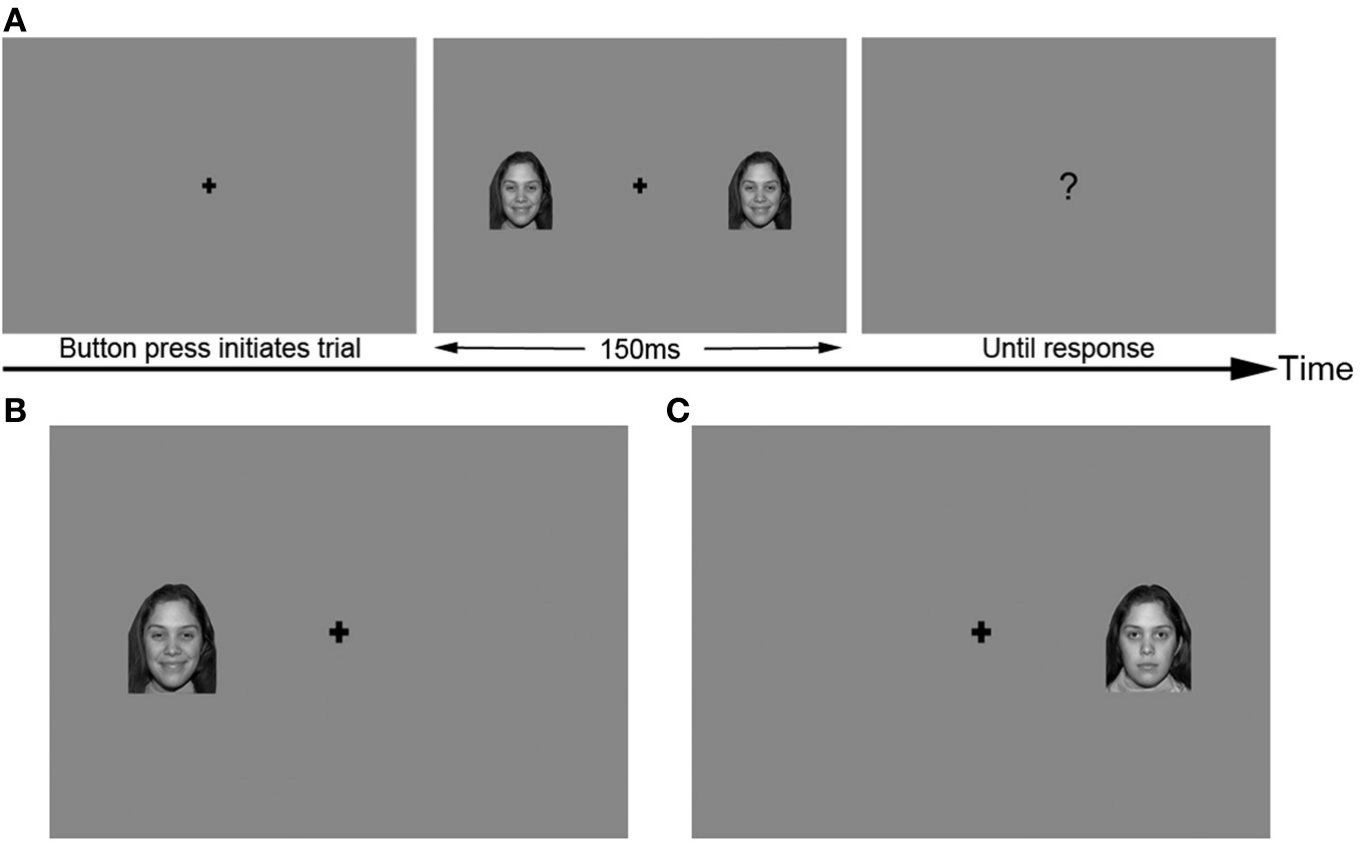
**Sample stimuli used in the primary identity and emotion tasks. (A)** Trial structure, with BVF stimulus. **(B)** LVF stimulus, happy expression. **(C)** RVF stimulus, neutral expression. Secondary conditions used the same trial structure and visual field locations of stimuli.

Images were presented using a PC computer running Windows 2000 XP and Neurobehavioral Systems Presentation® software (Version 10, build 07.03.06), and were displayed on a 21″ color CRT monitor at a distance of approximately 200 cm from the subject. All face images were grayscale and subtended a visual angle of 2.4 × 3.6 degrees. Images appeared at horizontal eccentricities of 2.9° visual angle from the center of the screen, and were vertically centered on the screen. Images appeared either in the RVF, LVF, or both visual fields simultaneously (BVF) at this eccentricity (Figure 1). A fixation cross appeared in the center of the screen throughout each trial. All stimuli were presented against a medium gray background.

### Procedure

#### Familiarization training

In order to allow subjects to complete the identity and emotion experimental tasks using the same set of stimuli, subjects were first familiarized with the stimuli by learning arbitrary English names for the eight “familiar” NimStim models included in the experiment. Subjects viewed each model's face five times, with five different expressions (happy, angry, sad, surprised, and disgusted), above their assigned names (Jill, Ann, Meg, Kate, Bill, Dave, Jeff, Tom). Each face and name remained on the screen for 4000 ms, and the faces were presented in random order. Subjects were then tested on their knowledge of the models' names by viewing each of the eight models' faces one at a time (posing a neutral expression), and choosing the correct name by pressing one of eight labeled keys on a keyboard. If subjects did not identify the correct name for every model, they repeated the familiarization and were tested again. All subjects who repeated the familiarization procedure obtained 100% accuracy on the second attempt. Thus, at the end of the familiarization, all participants had seen each model posing each facial expression and had correctly identified all models in the set.

All participants completed this familiarization training as part of a larger battery of face perception studies in our laboratory, and completed the current study within 2 weeks (range 1–14 days) of learning the faces. To remind participants of the faces that they learned during the familiarization training, all participants were shown the eight familiar faces (posing neutral expressions) and their corresponding names twice more in random order immediately before beginning the experimental trials. This procedure was intended to ensure that these models remained familiar to subjects, as compared to the unfamiliar models.

#### Experimental trials

After completing the familiarization training, subjects completed the experimental trials. At the start of each trial, a fixation cross appeared alone on the screen, and subjects were instructed to keep their eyes fixated on the cross throughout the entire trial. Subjects used a USB game pad (Gravis Game Pad Pro) to make their responses, which were recorded by the Presentation software. After subjects pressed a button on the game pad to initiate the trial, a face appeared randomly either in the RVF, LVF, or BVF for 150 ms, followed by a question mark. Subjects were asked to respond as quickly as possible after the question mark. Shifts in gaze were discouraged by presenting RVF, LVF, and BVF trials in a random order, and by presenting faces for only 150 ms (typically not long enough to execute a saccade to an unpredictable location). In addition, participants were monitored using a video feed to detect visible eye movements during the task.

Subjects completed four blocks of experimental trials:

Two blocks contained the primary experimental conditions. The primary emotion condition contained the eight familiar and eight unfamiliar NimStim models, posing happy and neutral expressions. Each of the 32 faces was presented in each of three visual field locations: RVF, LVF, and BVF, for a total of 96 experimental trials. The trials were randomly shuffled by the Presentation software, so that each participant viewed the stimuli in a different random order. For each image, subjects were asked to answer the yes/no question “Is the face happy?”

In the primary identity condition, the stimulus set was identical to that in the primary emotion condition, including the same eight familiar and eight unfamiliar models posing happy and neutral expressions, with each face appearing once in each visual field location, for a total of 96 trials. Again, trials were randomly shuffled, so that each participant viewed the stimuli in a different random order from one another and from the other experimental blocks. Subjects were asked to answer the yes/no question “Is the face familiar?”

The remaining two blocks contained secondary experimental conditions. In the secondary emotion condition, participants were presented with the same eight familiar and eight unfamiliar NimStim models as above, posing angry and neutral expressions. Again, each of these 32 faces was presented in each visual field location (RVF, LVF, and BVF) for a total of 96 trials, presented in a random order. In this block, subjects were asked to answer the question, “Is the face angry?”. This condition was added in order to determine whether our results would generalize to facial expressions other than happiness.

In the secondary identity condition, participants were presented with the eight *famous faces* (described above) and the same eight unfamiliar NimStim models as used in the primary conditions, posing happy expressions only. Because only one facial expression was used in this block of trials, each face appeared twice in each visual field location, to reach a total of 96 trials. In this block, subjects were asked to answer the question, “Is the person famous?”. This task examined whether level of familiarity would influence visual field effects observed during identity judgments. All of the deaf participants, and 10 of the 12 hearing participants completed the secondary conditions.

The order of the four blocks was counterbalanced by randomly assigning subjects to one of four possible orders, judging whether faces were Familiar/Famous/Happy/Angry; Famous/Familiar/Angry/Happy; Happy/Angry/Familiar/Famous; or Angry/Happy/Famous/Familiar. For all blocks, subjects made yes/no responses using the index fingers of both hands to press two buttons on a game pad simultaneously, in order to avoid a lateral bias in motor responses. Accuracy and reaction time (RT) were recorded.

#### Data analysis

Accuracy and RT were subjected to a three standard deviation outlier screen. Standard deviations were calculated within each experimental condition for each group, separately. As a result, one deaf participant's data were excluded from reaction time analyses due to slow responses in both identity conditions. Video monitoring indicated that participants were successful in maintaining fixation on the central fixation cross and no trials were rejected on the basis of lateral saccades.

We first examined visual field asymmetries for the primary experimental conditions, which contained identical stimulus sets but differing task demands. Accuracies and RTs for only the primary experimental tasks were each submitted to a repeated measures, mixed-model analysis of variance (ANOVA) with task (emotion vs. identity judgments) and visual field (LVF, RVF, BVF) as within-subject factors and group (deaf, hearing) as a between-subjects factor. Interactions between task and visual field were further examined by analyzing means for hearing and deaf groups in separate ANOVAs.

Next, we compared visual field asymmetries across groups in response to the primary and secondary experimental conditions. These analyses were meant to determine whether visual field effects observed using highly controlled stimuli in the primary conditions would remain when stimuli with different expressions or levels of familiarity were added to the analysis. Accuracy and reaction time across all four experimental tasks were compared in a repeated-measures ANOVA with task (emotion vs. identity), condition (primary vs. secondary), and visual field (LVF, RVF, BVF) as within-subjects factors, and group as a between-subjects factor. Interactions between task, condition, and visual field were further examined by analyzing means for hearing and deaf groups in separate ANOVAs.

## Results

### Primary conditions

Initial analyses examined the effect of task demands on visual field asymmetries in the two primary conditions, which presented the same set of familiar stimuli but required identity judgments in one block and emotion judgments in the other.

No overall differences in accuracy [*Group main effect: F*_(1, 22)_ = 0.358, *p* = 0.56] or reaction time [*F*_(1, 21)_ = 0.087, *p* = 0.77] between hearing and deaf groups were observed.

Participants across groups performed more accurately overall in the emotion (95% CI: 85.3 ± 2.5) than the identity task [95% CI: 81.4 ± 3.5; *Task main effect: F*_(1, 22)_ = 5.853, *p* = 0.024], There were no differences in overall RTs between tasks [*F*_(1, 21)_ = 2.306, *p* = 0.14].

A *Visual field main effect* for accuracy [*F*_(2, 44)_ = 3.272, *p* = 0.047] indicated that both groups were more accurate when faces were presented in both visual fields (95% CI: 85.7 ± 3.5), compared to presentations in the RVF (81.7 ± 3.1, pairwise comparison with BVF: *p* = 0.022), and LVF (82.6 ± 3.2, pairwise comparison marginal at *p* = 0.061), while accuracies in response to LVF and RVF presentations did not differ. A *visual field main effect* for reaction time [*F*_(2, 42)_ = 3.457, *p* = 0.041] indicated that subjects responded more slowly to faces in the RVF (95% CI:1485 ms ± 106) compared to LVF (95% CI:1426 ms ± 121, *p* = 0.01), while reaction times in response to BVF trials were intermediate (95% CI: 1434 ms ± 127) and did not significantly differ from RVF (*p* = 0.1) or LVF trials (*p* = 0.8).

**Figure 2:**
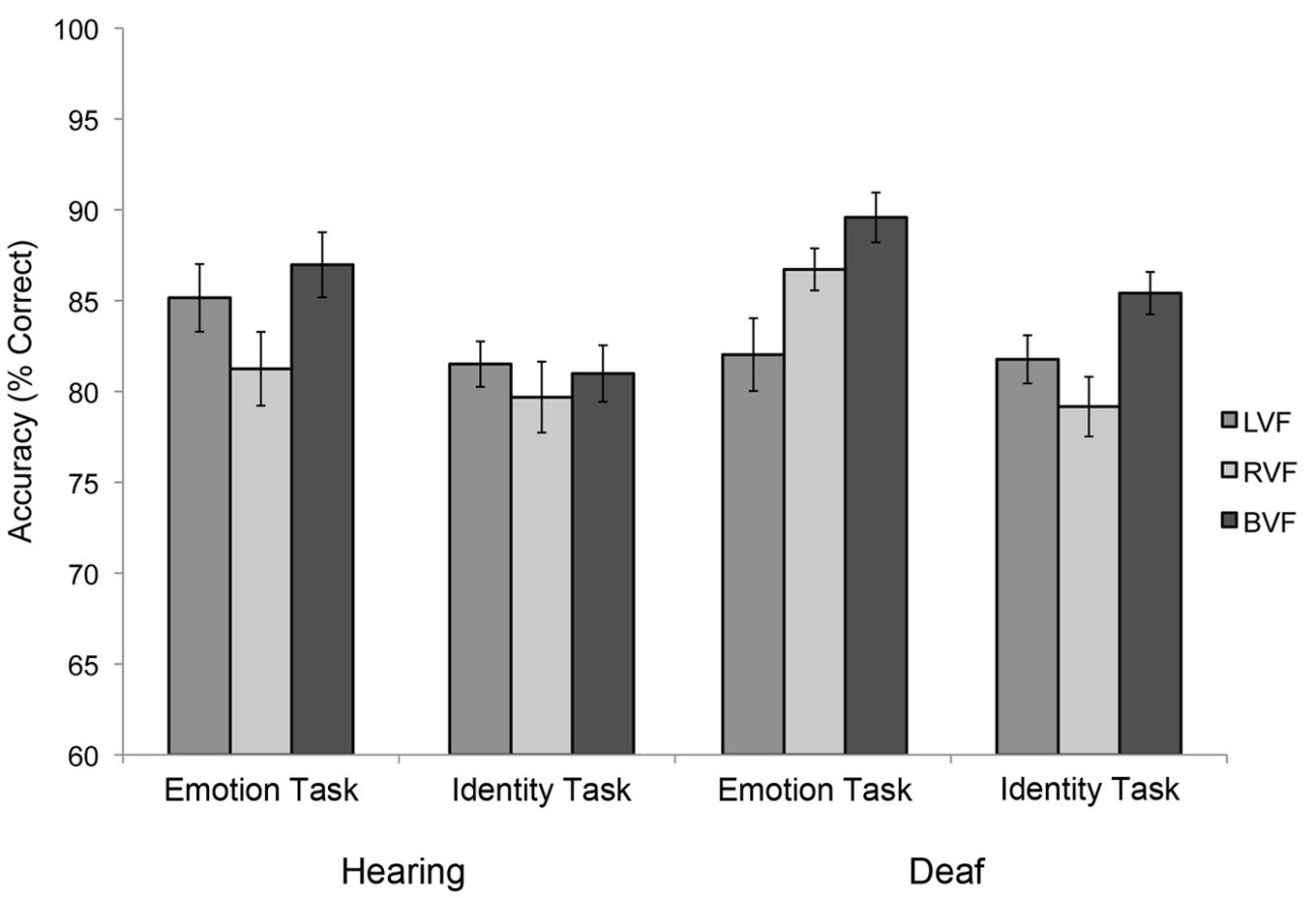
**Visual field asymmetries in the two subject groups, during the primary emotion task (“Is the face happy?”) vs. the primary identity task (“Is the face familiar?”), showing similar visual field biases across tasks for the hearing group, but opposite visual field biases between tasks for the deaf group**. Error bars represent the within-subjects standard error of the mean.

A *Task* × *Visual field* × *Group interaction* for accuracy was marginal [Figure 2; *F*_(2, 44)_ = 2.598, *p* = 0.086]. This interaction was explored in more detail by testing the two groups in separate ANOVAs with task (emotion, identity) and visual field as the within-subject factors. In these analyses, a *Task × Visual field interaction* was not significant for the hearing group [*F*_(1, 11)_ = 1.997, *p* = 0.2]. As shown in Figure 2, in both tasks hearing subjects were most accurate for BVF presentations, least accurate for RVF presentations, with LVF presentations intermediate, but differences between the three conditions failed to reach significance (*p*-values 0.5 to 0.8). No significant effects were found for reaction time.

By contrast, a marginally significant *Task* × *Visual field interaction* was observed for the deaf group [*F*_(2, 22)_ = 3.215, *p* = 0.06], revealing different visual field effects during emotion vs. identity judgments (Figure 2). During the identity task, deaf subjects were more accurate in response to BVF (95% CI: 85.4 ± 6.3) than RVF presentations (79.2 ± 5.4; pairwise comparison *p* = 0.031), with LVF intermediate (81.7 ± 5.3; marginal contrast with BVF: *p* = 0.078; non-significant contrast with RVF: *p* = 0.36). However, in the emotion task, deaf adults were more accurate in response to BVF (89.6 ± 4.8) than LVF presentations (82.0 ± 5.6; *p* = 0.039) with RVF intermediate (86.7 ± 4.6; non-significant contrast with BVF: *p* = 0.094; and LVF: *p* = 0.14). No significant task interactions were found for reaction time.

In sum, for the hearing group, visual field biases manifest as lowest accuracy for RVF compared to BVF presentations (with LVF intermediate and non-significantly different from either RVF or BVF) and this pattern did not appear to vary with task demands. Deaf subjects were also less accurate in response to RVF than BVF presentations when judging identity, but less accurate in response to LVF than BVF presentations when judging emotion. These numerical differences in accuracies were observed despite the fact that the stimuli in these blocks were identical. Only their responses in the emotion task appeared to differ from the pattern observed in hearing adults. These results suggest that deafness and/or ASL experience had only a small effect on visual field asymmetries, and specifically during emotion judgments.

### Primary vs. secondary conditions

We next examined whether these results would generalize to other stimuli. All of the deaf participants and ten of the hearing participants completed two additional experimental conditions with similar task demands, but slightly different stimuli, and visual field effects were again compared across emotion and identity tasks. In the secondary emotion condition, participants viewed the same familiar and unfamiliar NimStim models as shown in the primary conditions, but in this condition they posed neutral and angry expressions. In the secondary identity condition, half of the faces were images of famous celebrities, rather than the familiarized NimStim models.

No overall differences in accuracy [*Group main effect: F*_(1, 20)_ = 0.006, *p* = 0.94] or reaction time [*F*_(1, 19)_ = 0.002, *p* = 0.97] between hearing and deaf groups were observed. In the analyses described below, no main effects or interactions were observed for reaction time, therefore reaction time will not be discussed further.

A *Task main effect* across groups indicated that participants performed more accurately when making identity (84.08 ± 3.32) rather than emotion judgments [81.09 ± 2.44; *F*_(1, 20)_ = 4.484, *p* = 0.047]. This effect was significant within the deaf group [*F*_(1, 11)_ = 12.091, *p* = 0.005; 95% CI for identity tasks, 85.24 ± 4.48, for emotion tasks, 80.12 ± 3.29] but not within the hearing group [*F*_(1, 9)_ = 0.113, *p* = 0.74; identity tasks 82.92 ± 4.91, emotion tasks 82.06 ± 3.6]. However, no *Task* × *Group interaction* was found [*F*_(1, 20)_ = 2.285, *p* = 0.146].

A *Visual field main effect* was observed across groups [*F*_(2, 40)_ = 6.871, *p* = 0.003], reflecting higher accuracy in response to BVF (85.45 ± 3.15) than both RVF (80.43 ± 3.05, *p* = 0.003) and LVF presentations (81.87 ± 2.86, *p* = 0.034), which did not differ from one another (*p* = 0.99). A *Visual field main effect* was also significant within the hearing and deaf groups individually [*Hearing: F*_(2, 18)_ = 3.584, *p* = 0.049; *Deaf: F*_(2, 22)_ = 4.070, *p* = 0.031], and direct comparisons between groups did not reach significance [*Visual field × Group: F*_(2, 40)_ = 0.927, *p* = 0.40].

A *Task × Condition interaction* was observed both across groups [*F*_(1, 20)_ = 75.223, *p* < 0.001] and within each group individually [*Hearing: F*_(1, 9)_ = 9.913, *p* = 0.012, *Deaf: F*_(1, 11)_ = 108.3, *p* < 0.001]. Both groups responded more accurately when judging happy expressions in the primary emotion condition (hearing 95% confidence interval 84.69 ± 4.64, deaf 86.11 ± 3.48) as opposed to angry expressions in the secondary emotion condition (hearing 79.44 ± 4.43, deaf 74.13 ± 4.08; pairwise comparisons between conditions, *Hearing: p* = 0.013, *Deaf: p* < 0.001). A *Task* × *Condition* × *Group interaction* [*F*_(1, 20)_ = 10.925, *p* = 0.004] revealed that only the deaf group judged famous faces in the secondary identity condition (hearing 84.38 ± 5.76, deaf 88.37 ± 4.41) more accurately than familiar faces in the primary identity condition (hearing 81.46 ± 7.24, deaf 82.12 ± 4.87; pairwise comparisons, *Hearing: p* = 0.273; *Deaf: p* = 0.004). However, in a direct comparison, the deaf and hearing groups did not differ from one another in either condition (*Familiar/unfamiliar condition: p* = 0.86, *Famous/unfamiliar condition: p* = 0.23).

A *Task* × *Visual field* × *Group interaction* did not reach significance [*F*_(2, 40)_ = 1.463, *p* = 0.24]. However, when the groups were analyzed individually, the hearing group showed no *Task × Visual field interaction* for accuracy [*F*_(2, 18)_ = 0.469, *p* = 0.633], while the deaf group again showed a marginal *Task* × *Visual field interaction* [*F*_(2, 22)_ = 3.087, *p* = 0.06]. Pairwise comparisons indicated that across the two identity conditions, the deaf group showed no evidence of any visual field effects, responding with similar accuracy to BVF (95% confidence interval 87.24 ± 4.82), LVF (84.77 ± 4.48) and RVF trials (83.72 ± 4.91; pairwise comparisons, *p*'s > 0.15). By contrast, across the two emotion conditions, the deaf group responded more accurately for BVF (83.7 ± 3.9) than LVF presentations (77.3 ± 4.5, *p* = 0.007), with RVF intermediate (79.3 ± 4.4; marginal contrast with BVF: *p* = 0.076, non-significant contrast with LVF: *p* = 0.99).

Finally, a *Task* × *Condition* × *Visual field interaction* was not significant overall [*F*_(2, 40)_ = 1.43, *p* = 0.25], or within the hearing group [*F*_(2, 18)_ = 0.78, *p* = 0.47] but was marginal for the deaf group [*F*_(2, 22)_ = 2.97, *p* = 0.07]. Pairwise comparisons indicated that the deaf group's higher accuracy in response to famous than unfamiliar faces was significant for stimuli presented in the RVF (familiar/unfamiliar vs. famous/unfamiliar: *p* = 0.001), marginal for BVF presentations (*p* = 0.07), and not significant for LVF presentations (*p* = 0.1).

In summary, similar patterns of responses to stimuli presented in the RVF, LVF, or BVF were observed in the primary experimental conditions, which contained identical models and expressions, as well as conditions that presented either different facial expressions or individuals that were more familiar to the subjects. Hearing participants consistently performed least accurately for RVF presentations, regardless of stimuli or task demands. While accuracies were significantly lower for RVF than BVF, numerical differences between RVF and LVF did not reach significance. Deaf participants produced a similar pattern with no significant differences when making identity judgments, but when making emotion judgments they were significantly less accurate in response to LVF than BVF presentations.

## Discussion

Deaf signers extract both linguistic and affective input from faces during everyday communication, and must make rapid discriminations in facial expressions in order to do so. The current study was designed to examine whether this increased reliance across the lifespan on facial information for communication reduces the typical LVF bias during non-linguistic face perception tasks, and whether this effect would be specific to judgments of emotion. Participants were presented with a set of stimuli containing both variations in identity (with some familiar faces and some unfamiliar) and emotion (with some happy faces and some neutral), and were asked to judge either the familiarity or the expression of those faces when presented in the RVF, LVF, or both. Both participant groups were most accurate in responding to stimuli presented in both visual fields. Hearing participants responded least accurately to RVF presentations across task demands and stimulus properties, a pattern similar to the LVF bias documented in previous studies (Rhodes, 1985; Schweinberger et al., 2003), although unlike in previous studies, direct comparisons of RVF and LVF presentations did not reach significance. Accuracies in the RVF trials were, however, significantly lower than those in BVF trials.

Several methodological issues might account for the attenuation of the LVF bias that was observed in the hearing group for all tasks and for the deaf group in identity tasks. Although participants were instructed to maintain their gaze on the central fixation cross throughout each trial, we did not directly track participants' gaze during this study. Therefore, if participants shifted their gaze during a trial, it is possible that the stimuli did not fall within the right or left peripheral visual field. However, given that the stimuli appeared in unpredictable locations, and remained on the screen for only 150 ms, it is unlikely that participants would have been able to execute saccades to the faces. Further, video monitoring throughout data collection sessions indicated that participants were compliant with instructions and did not saccade within trials. Similar methods have been used successfully in previous studies (Young et al., 1985; Schweinberger et al., 2003). However, future studies could use eye-tracking measures to fully ensure that faces remain in the right or left peripheral visual field throughout the experimental trials.

Qualities of the tasks or the stimuli in the current study (which were tightly controlled and highly similar) may also have minimized the typical LVF bias in hearing subjects and in deaf subjects during identity judgments. For example, it is possible that participants may have relied on individual features of the face to discriminate expressions or individuals, a strategy which might reduce the LVF bias. In addition, half of the faces in each experimental block were familiar to participants either because they were trained or because they were famous. Previous research has documented largest visual field effects for tasks involving judgments of unfamiliar faces (Rhodes, 1985), and has found bilateral advantages (demonstrated by higher accuracies for BVF presentations than either RVF or LVF) for familiar but not unfamiliar faces (Mohr et al., 2002; Schweinberger et al., 2003; Baird and Burton, 2008). These studies have also observed small or non-significant differences in accuracy between LVF and RVF presentations when subjects judged familiar faces (Mohr et al., 2002; Schweinberger et al., 2003). Researchers have argued that the semantic information attached to familiar and famous faces may increase the involvement of the left hemisphere, potentially reducing the LVF bias and resulting in a bilateral advantage.

Previous examinations of the respective influences of emotion and identity processing on visual field biases were limited because they conflated familiarity and identity. For example, Schweinberger et al. (2003) compared accuracy and reaction time in an emotion and an identity task, when faces were presented in the RVF, LVF, or BVF simultaneously. Their emotion task included only unfamiliar faces, while their identity task included familiar and unfamiliar faces. A significant LVF bias was observed in their emotion task, but no significant differences between RVF and LVF presentations were observed in their identity task. Similarly, Mohr et al. (2002) found a bilateral advantage in an identity task with familiar faces, but no difference in accuracy between LVF and RVF presentations. In the current study, participants observed familiar and unfamiliar faces across emotion and identity tasks and produced a similar advantage for BVF presentations. This suggests that our choice to carefully control for familiarity in the stimuli across the identity and emotion tasks may have reduced the typical LVF bias and resulted in a BVF advantage, although this hypothesis remains a topic for further study. More studies with within-subject designs and larger sample sizes are necessary to determine what combinations of stimulus qualities and task demands elicit different laterality patterns. Nevertheless, the patterns we observed across visual fields in the hearing group were consistent with those reported in previous studies, and allowed us to examine the effects of task demands within the hearing and deaf groups (the primary purpose of the current study).

Direct comparisons of the hearing and deaf groups did not reveal statistically significant differences, but within-group analyses showed that mean accuracy across visual fields appeared to vary with task demands only within the deaf group. Only deaf participants showed significantly lower accuracy for RVF than BVF during the primary identity condition, but marginally lower accuracy for LVF than BVF during the primary emotion condition, despite the fact that the stimuli in these conditions were completely identical. A similar pattern was observed across the primary and secondary emotion tasks, in which participants detected either happy or angry faces, respectively. These results extend the findings of previous studies of face perception in the deaf, which report reduced or reversed visual field asymmetries during perception of emotional faces (Vargha-Khadem, 1983; Szelag and Wasilewski, 1992; Szelag et al., 1992; Corina et al., 1999). These studies were often limited by the use of different stimuli across tasks, by the inclusion of linguistic information in the stimuli (e.g., linguistic facial expressions or manual signs), or by task demands that involved linguistic processing (Corina, 1989; Vargha-Khadem, 1983). The current study was designed to control for these issues by presenting faces posing varying emotional expressions in both tasks, and by manipulating task demands to require explicit attention either to expression or to identity. With these constraints, visual field biases in the deaf group's responses to faces were influenced by task demands, although the differences observed across visual fields were fairly small. Nonetheless, from our observations of this small sample, we can conclude that these modulations in visual field biases in the deaf group were dependent on explicit attention to the emotional expressions of the faces, and not the mere presence of emotion information in the stimuli.

Generalizability of our findings was examined by having subjects complete two secondary conditions that involved the same task demands, but different stimuli than the primary experimental conditions. While in some cases the secondary conditions were easier than the primary conditions, they elicited similar patterns of responses across visual fields. In the hearing group, RVF presentations always elicited lowest accuracies across tasks and conditions, differing significantly from BVF presentations but not from LVF presentation. By contrast, the deaf group showed patterns of responses across visual fields that were modulated by task demands across stimulus conditions. Thus, the visual field differences we observed do not appear to be specific to either stimulus set employed in the current study.

The direction of numerical differences in accuracy across visual fields did not differ between the two emotion conditions, despite the use of a positive valence emotion in one condition, and a negative valence in the other. These findings are in contrast to reports that positive and negative emotions may elicit opposite visual field asymmetries (see Adolphs, 2002, for a discussion). Instead, they support the hypothesis that a tendency toward a RH/LVF bias is a generally-observed pattern in hearing adults during the perception of expressive faces. However, the present study is limited in that we examined visual field asymmetries for only two expressions (one positive valence, and one negative). Further, we did not explicitly control arousal or intensity of the emotional expressions; we chose closed-mouth, non-extreme versions of happy and angry expressions available in the NimStim set. Therefore, it is possible that different levels of intensity of these emotions could alter the visual field biases that we observed.

Together, the results of the current study indicate that visual field biases in hearing non-signers did not vary with either task demands or stimulus properties. In contrast, deaf signers showed varying response patterns across tasks. RVF presentations elicited the poorest performance for both groups when judging identity, even though emotional expression varied in the stimuli, and for hearing subjects when judging emotion. However, when judging emotion, the deaf group instead responded numerically least accurately to LVF presentations, the opposite of the predicted pattern in a typical LVF bias. This result is important because it indicates that, for deaf signers, the visual field bias for face perception depends not simply on stimulus properties but on the specific direction of attention to expressive information on the face. Although these task variations in deaf signers were modest, and the interactions observed between task demands and visual field effects were only marginally significant, similar patterns were observed across two different sets of stimuli. Follow-up studies with a larger sample size and a greater number of trials may help to verify the effects observed here. It would be worthwhile to conduct additional studies that systematically vary stimulus qualities while holding task demands constant, or vice versa. Such larger scale studies may help to determine the extent to which visual field biases are variable in deaf signers under different conditions.

Previous studies have found variations in visual field biases and accompanying hemispheric asymmetries in deaf signers in different tasks. For example, neuroimaging studies have suggested that deaf signers may recruit left hemisphere structures to different degrees in face perception tasks, depending on the type of information being extracted from the face. The FG and STS in both the left and right hemispheres are involved in face processing for both hearing non-signers and deaf signers (McCullough et al., 2005). However, aspects of face perception that are inherently tied to ASL communication, such as rapid, on-line judgments of facial expression, may increase the engagement of those left hemisphere regions in deaf signers, resulting in either a lack of visual field bias, or a RVF bias, depending upon the stimuli and task.

This hypothesis is in line with previous evidence that visual field and hemispheric asymmetries in the deaf are altered during the perception of both emotional and linguistic expressions (Vargha-Khadem, 1983; Szelag and Wasilewski, 1992; Szelag et al., 1992; Corina et al., 1999; McCullough et al., 2005) and that these effects may be influenced by task demands (Corina, 1989). The current results are also consistent with evidence from our own lab that hemispheric asymmetries of face-sensitive event-related potentials in deaf signers are not altered during perception of neutral faces or during same/different face discriminations (Mitchell et al., 2013). Follow-up studies in our laboratory are currently examining face-sensitive neural responses during identity and emotion tasks that utilize identical stimuli, in order to determine whether hemisphere asymmetries at early stages of face perception are also dependent on attention to emotional expression in deaf signers.

A final limitation of this study is the lack of separability of effects due to deafness itself from those due to lifelong ASL use. Our results indicate that differences in the laterality of face perception between hearing non-signers and deaf signers may not be limited to tasks that directly involve ASL comprehension, but may extend to other tasks where facial expression is relevant. However, it is possible that deafness itself—independently from ASL experience—can drive the effects that we observed in the deaf signing group. Studies of hearing native signers who are born into deaf families, and therefore acquired ASL as their first language, can help to differentiate the impact of native ASL experience from that of deafness itself. Studies of motion perception have recruited members of this group and have concluded that ASL experience did not elicit enhanced processing of visual motion but did drive a shift in laterality toward the left hemisphere/RVF (Neville and Lawson, 1987a,b; Bosworth and Dobkins, 1999, 2002a). The one published study of hemispheric asymmetry for face processing in hearing native signers reported non-identical effects of ASL and deafness (Emmorey and Mccullough, 2009). Native ASL acquisition in and of itself did not result in a left hemispheric asymmetry for face processing and only reduced the typical right hemispheric asymmetry when facial expressions were linguistic in nature. The authors hypothesized that the effects of deafness on the broader organization of face processing is linked to increased local featural processing (Emmorey and Mccullough, 2009), specifically of the mouth area, either for lipreading or for encoding ASL gestures of and around the mouth (see McCullough and Emmorey, 1997; Letourneau and Mitchell, 2011; Mitchell et al., 2013). This increased local processing, in turn, increases the engagement of the LH into the general domain of face processing. Thus, it appears that there is an additive effect; native ASL use increases the engagement of the left hemisphere for linguistic purposes, but not enough to result in a left hemispheric asymmetry, while deafness imposes additional attention to the bottom half of the face, ultimately resulting in a left hemispheric asymmetry and a reduction in the typical LVF bias for face processing. The current findings suggest that this need to process information around the mouth area imposes sufficient pressure on the face processing system in deaf signers that the reduction in the LVF bias can be observed in the absence of linguistic information or analysis if attention is directed toward emotional information. We are currently examining hemispheric asymmetries of face-selective event-related potentials in these same participants, which will allow for a deeper analysis of the correlations between those asymmetries and visual field biases in face processing and the mechanisms that determine their dynamic recruitment in auditory deprivation and lifelong sign experience.

### Conflict of interest statement

The authors declare that the research was conducted in the absence of any commercial or financial relationships that could be construed as a potential conflict of interest.
